# Effect of burosumab conversion on calciuria and nephrocalcinosis in children with XLH: A real-world cohort study

**DOI:** 10.1016/j.bonr.2025.101877

**Published:** 2025-09-06

**Authors:** Guido Filler, Harry Chandrakumaran, Funmbi Babalola, Dougenie Emile, Shih-Han Susan Huang, Robert Stein

**Affiliations:** a Department of Pediatrics, Schulich School of Medicine and Dentistry, University of Western Ontario, London, Canada; b Department of Medicine, Schulich School of Medicine and Dentistry, University of Western Ontario, London, Canada; c Lilibeth Caberto Kidney Clinical Research Unit, London, Ontario, Canada; d Children's Health Research Institute, London, Canada; e Hospital for Sick Children, University of Toronto, Toronto, Canada; f Department of Pediatrics, Hôpital Universitaire de Mirebalais, Port-au-Prince, Haiti; g University of Western Ontario, London, Canada

**Keywords:** Burosumab, Hypercalciuria, Nephrocalcinosis, PHEX gene mutation, x-Linked hypophosphatemic rickets

## Abstract

**Background:**

Burosumab, a monoclonal antibody to fibroblast growth factor 23 (FGF23), is effective for X-linked hypophosphatemic rickets (XLH). Renal effects, particularly calciuria and nephrocalcinosis, remain incompletely characterized.

**Methods:**

In this retrospective cohort, 13 children with genetically confirmed XLH (7 females, 6 males; 0.6–16.3 years) were evaluated after conversion from conventional therapy to burosumab. Longitudinal changes in serum phosphate, TmP/GFR, TRP, 1,25(OH)₂D, intact parathyroid hormone (PTH), and urinary calcium/creatinine (Ca:Cr) were assessed. Hypercalciuria was defined using age-specific SI thresholds; Ca:Cr was also expressed as ×ULN (Ca:Cr divided by the age-specific upper limit).

**Results:**

Burosumab improved serum phosphate (*p* < 0.001), TmP/GFR (p < 0.001), and TRP (*p* = 0.007). Despite normalized phosphate handling, two patients developed de novo nephrocalcinosis. No child was hypercalciuric at washout or Day 14; two had ≥1 episode later. A repeated-measures analysis of log10(×ULN) showed no overall time effect from washout to Day 56 (F(4,29) = 1.66, *p* = 0.185). A larger decline in PTH correlated with higher Ca:Cr (*p* < 0.05), whereas 1,25(OH)₂D did not.

**Conclusion:**

Burosumab improves phosphate homeostasis in children with XLH, but a minority may develop hypercalciuria and nephrocalcinosis, potentially linked to PTH suppression. Vigilant biochemical and ultrasound monitoring—particularly early after conversion—and consideration of prophylaxis in high-risk cases are advisable.

## Highlights

• Burosumab normalized phosphate handling in a pediatric XLH cohort.
• Two children developed de novo nephrocalcinosis after conversion.
• Two children had ≥1 episode of hypercalciuria during follow-up.
• Larger iPTH declines were tracked with higher urinary Ca:Cr ratios.
• Early monitoring and cautious titration may help reduce the risk of calcification.

## Graphical abstract

**Figure:**
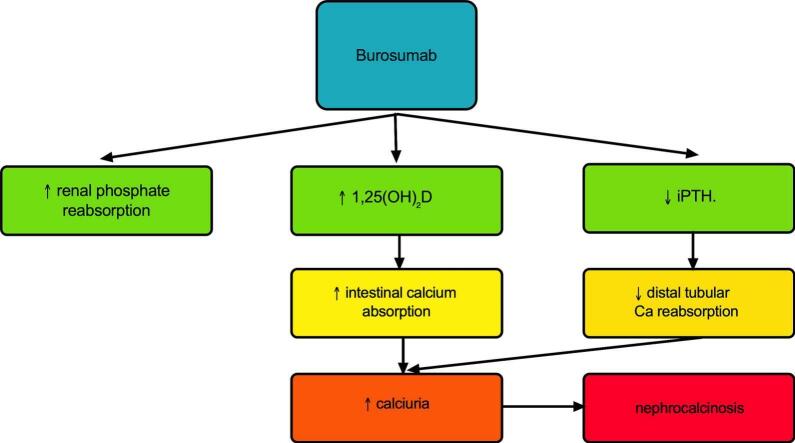

## 1 Background

X-linked dominant hypophosphatemic rickets (XLH, OMIM 307800) is a rare heritable disease due to mutations of the *PHEX gene*, which leads to renal phosphate wasting and subsequently to hypophosphatemia; rickets with bone deformities; short stature; dental anomalies; normal serum calcium; urinary calcium excretion is typically low-to-normal; normal or low serum level of vitamin D (1,25(OH)_2_D, or calcitriol); and increased activity of serum alkaline phosphatase (Gohil and Imel, 2019). While many untreated patients have normal PTH, elevations can occur even prior to phosphate therapy. The *PHEX* gene encodes for the human phosphate-regulating endopeptidase, which belongs to the type II integral membrane zinc-dependent endopeptidase family (Filisetti et al., 1999). Mutations in the PHEX gene are associated with elevated intact FGF23 levels, though the exact mechanism—whether due to altered degradation or increased expression—remains incompletely understood (Filisetti et al., 1999). Traditional therapy consists of oral phosphate supplementation and calcitriol therapy, which often leads to hypercalciuria, hyperparathyroidism, and nephrocalcinosis (Ali et al., 2025). Yet, rickets rarely completely resolves, and short- and long-term complications have been reported during conventional therapy, including diarrhea, hypercalciuria, nephrocalcinosis (prevalence up to 47 % in treated patients), and adult hypertension (Arango Sancho, 2020; Colares Neto et al., 2019; Raeder et al., 2008). Burosumab is a fully human monoclonal antibody that targets FGF23 and is approved for XLH in many countries (e.g., FDA, EMA). In pediatrics, randomized open-label data show superiority vs conventional therapy (Imel et al., 2019), whereas adult trials were placebo-controlled and showed improvement vs placebo (Insogna et al., 2018; Portale et al., 2019). The effect of switching pediatric patients from conventional treatment to burosumab on hypercalciuria is understudied.

## 2 Methods

This was a retrospective cohort study of all XLH children treated with burosumab in a single tertiary center. Patients were eligible for inclusion if their treatment commenced from January 2020 to May 2022. The study was approved by the research ethics committee of the University of Western Ontario (Project ID 118344, last modified 02/May/2022). Clinical data, burosumab doses, anthropometry, and laboratory results were extracted from the electronic medical records system and entered into a spreadsheet securely stored on a protected and encrypted hospital network drive. Burosumab was started after a one-week washout from conventional therapy. Patients were monitored initially every two weeks for 42 days after the switch and then at 3-month intervals. For laboratory testing, fasting was not required, and urine samples were spot samples. Not all the patients received their burosumab doses in the clinic and thus the laboratory testing was not always done at the end of a dosing interval. Dose adjustments were therefore based on alkaline phosphatase levels rather than phosphate concentrations. Genetic studies were extracted from the paper charts. Only anonymized data were recorded; a separate spreadsheet with study numbers was available only to GF.

We analyzed available data from the electronic medical records of all cases using descriptive statistics. Analysis was performed in GraphPad Prism version 10 for Mac, San Diego, California, USA, licensed to GF. Burosumab doses were calculated in mg/kg. Pediatric iPTH reference intervals vary widely with age, but all values were interpreted within our institutional laboratory's appropriate age-specific reference range. The ratio of the tubular maximum reabsorption rate of phosphate to glomerular filtration rate (TmP/GFR) was calculated according to Brodehl et al. (1988). The severity of the rickets was assessed using the Thacher rickets severity score by a single radiologist at our institution (Thacher et al., 2000).

Urine calcium/creatinine (Ca:Cr) and hypercalciuria (SI). All Ca:Cr values are reported in mmol/mmol. Hypercalciuria was defined by age-specific thresholds (×(Upper limit of normal) ULN > 1.0) using institutional pediatric reference intervals (0–6 mo >2.0; 7–12 mo >1.5; 1–3 y > 1.5; 3–5 y > 1.1; 5–7 y > 0.8; >7 y > 0.8 mmol/mmol) (Marra et al., 2019). For children who crossed age categories during follow-up, the threshold corresponding to the specimen date was applied. For figures, Ca:Cr values are displayed as ×ULN = Ca:Cr ÷ age-specific ULN (dimensionless), enabling a single dashed reference line at 1.0 across ages.

We used simple statistics to describe the data. We used the D'Agostini Pearson omnibus test for normality and the parametric paired *t*-tests or the non-parametric Wilcoxon tests where applicable. For the correlation analysis we used the Spearman rank correlation method as data were not normally distributed. For the drop of the iPTH, we calculated the difference of the average of up to 4 pre-Burosumab treatment results with the four subsequent measurements after starting burosumab. For urinary calcium, we analyzed log10(×ULN) with a patient-matched repeated-measures model (time categorical: Washout, Days 14, 28, 42, 56), reporting the global time effect and pre-specified contrasts vs Washout as ratios of geometric means (back-transformed) with 95 % CIs and Holm adjustment; analyses were exploratory without a global multiplicity correction.

## 3 Results

### 3.1 Patient cohort and baseline characteristics

We treated 13 children (7 female) with XLH using burosumab. The median age at treatment initiation was 3.7 years (range 0.6–16.3). Eleven of the 13 patients had been previously treated with conventional therapy and underwent a 7-day washout before burosumab initiation. Of note, Patients 1, 2, and 8–11 were cousins, and Patients 6 and 7 were siblings. Prior to treatment, all patients exhibited Thacher rickets severity scores ranging from 2 to 10 and TmP/GFR values well below the 2.5th percentile. The mean serum phosphate at the end of the washout period was 0.77 ± 0.20 mmol/L, and the mean TmP/GFR was 0.66 ± 0.20 mmol/L. Patient-level genetic and clinical characteristics are summarized in Table 1.

**Table 1:** Demographics, genetics, and baseline parameters of the patients included in this study. PO_4_ = serum phosphate, TmP/GFR = mean ratio of tubular maximum reabsorption rate of phosphate to glomerular filtration rate, F/U = follow-up.

Study ID	*PHEX* gene defect	Sex	Serum PO_4_ after washout [mmol/L]	TmP/GFR after washout [mmol/L]	Duration of conventional therapy [years]	Age at start of burosumab [Years]	Age at last F/U [years]
1	c.1753G>A (p.G585R)	M	0.98	0.73	1.0	1.2	5.2
2	c.1753G>A (p.G585R)	M	0.73	0.74	2.9	3.3	7.2
3	c.1645+1G>A (splice site), intron 15-16	F	0.50	0.38	1.4	8.4	12.3
4	4.36 kb exon 2 deletion (GRCh37/hg19)	M	0.52	0.45	0.3	2.7	6.8
5	c.610_612delATC (p.I204del)	F	0.79	0.72	9.0	11.0	14.1
6	Complete exon 12 and 13 deletion	M	0.61	0.56	4.5	5.1	9.1
7	Complete exon 12 and 13 deletion	M	0.60	0.49	7.6	7.8	11.8
8	c.1753G>A (p.G585R)	F	1.04	0.83	0.9	1.3	4.9
9	c.1753G>A (p.G585R)	F	1.00	0.96	0.1	0.6	4.2
10	c.1753G>A (p.G585R)	F	0.97	0.91	3.4	3.7	7.3
11	c.1753G>A (p.G585R)	F	1.12	0.87	0.5	1.0	2.1
12	535 kb loss of Xp11.aa arr[hg19]Xp22.11(22228334_22763463)x0	M	NA	NA	NA	16.3	18.5
13	c.1208G>A, p.Trp403*	F	0.71	0.47	0.7	6.0	8.1
Mean			0.77	0.66	2.9	5.2	8.6
Standard Deviation			0.20	0.20	3.0	4.7	4.5
Median			0.73	0.72	1.4	3.7	7.3
Min			0.50	0.38	0.1	0.6	2.1
Max			1.04	0.96	9.0	16.3	18.5

NA- Not available.

Three patients (1, 2, 3) had medullary nephrocalcinosis at baseline. Patient 11 developed de novo nephrocalcinosis after conversion. Patient 10 developed new punctate echogenic foci not present at baseline, consistent with possible early de novo nephrocalcinosis; one case subsequently resolved on follow-up ultrasound.

### 3.2 Conventional therapy parameters

The mean daily alfacalcidol dose was 0.031 ± 0.020 μg/kg, and the mean sodium phosphate dose was 60.4 ± 36.7 mg/kg. After the washout period, TmP/GFR dropped further to 0.63 ± 0.19 mmol/L.

### 3.3 Burosumab dosing and treatment response

The starting dose of burosumab was 0.84 ± 0.19 mg/kg subcutaneously every 14 days. At the last follow-up (mean duration 3.3 ± 0.9 years), the mean body weight had increased to 24.6 ± 10.5 kg. The average burosumab dose at that time was 1.50 ± 0.63 mg/kg.

Treatment resulted in significant improvements in phosphate metabolism:

• **Serum phosphate** increased from 0.77 ± 0.20 to 1.09 ± 0.24 mmol/L (*p* = 0.0001845),
• **TmP/GFR** increased from 0.63 ± 0.19 to 1.01 ± 0.22 mmol/L (*p* = 8.53 × 10^−5^),
• **TRP** improved from 0.71 ± 0.21 to 0.93 ± 0.04 (*p* = 0.0069).

**Fig. 1:**
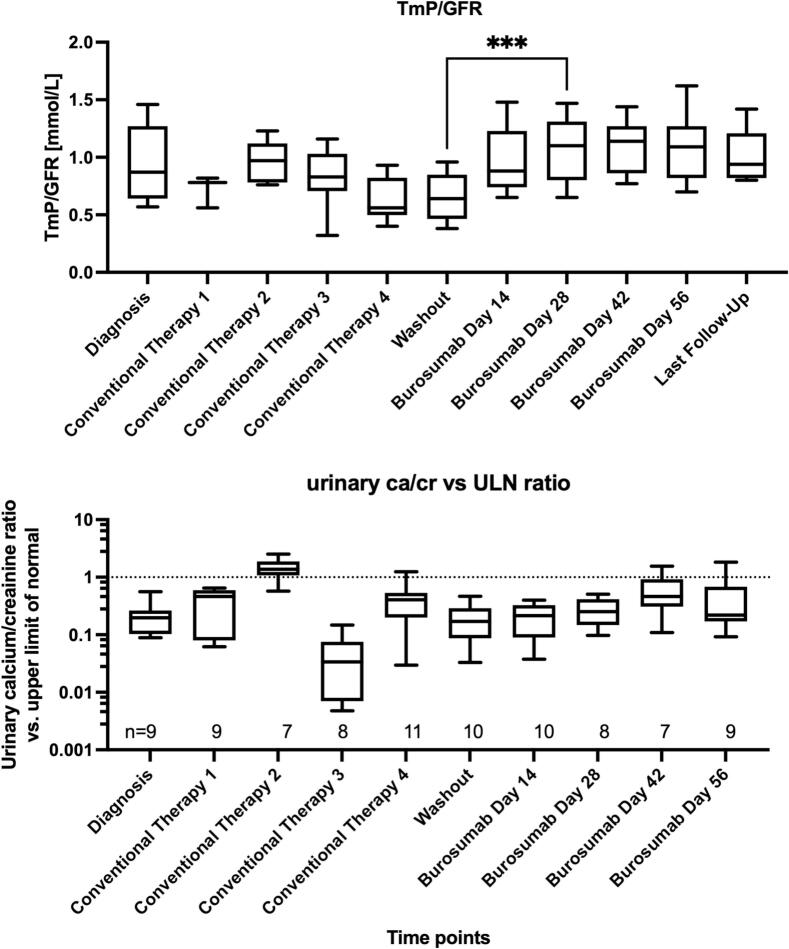
(A) The effect of switching to burosumab on TmP/GFR. (B) Urine calcium/creatinine expressed as ×ULN = Ca:Cr ÷ age-specific ULN (dimensionless; log10 scale). The dashed line at 1.0 denotes the hypercalciuria threshold. Boxes show median, IQR; whiskers 5–95 %. *** *p* < 0.001.

These changes are illustrated in Fig. 1.

### 3.4 Calcium metabolism, PTH suppression, and nephrocalcinosis

**Fig. 2:**
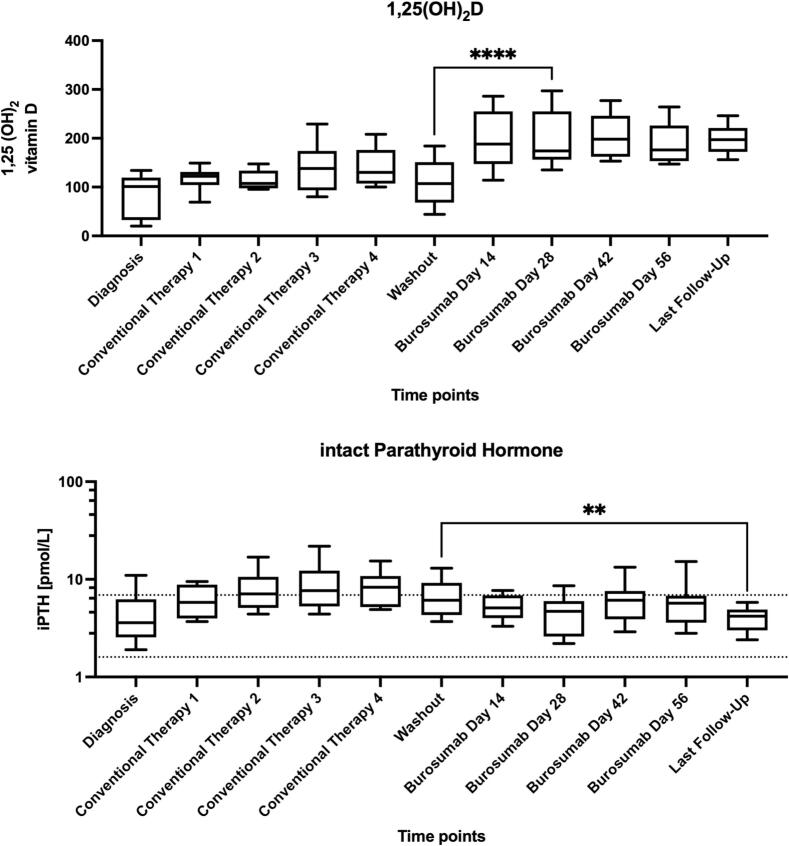
(A) The effect of switching to (or starting) burosumab on 1,25(OH)_2_D levels and (B) Intact Parathyroid Hormone. ** means the p-value was less than 0.01, and **** means the p-value was less than 0.0001.

Burosumab therapy was associated with a significant rise in 1,25(OH)₂D levels and a notable suppression of intact PTH, as shown in Fig. 2. Two patients had ≥1 episode of hypercalciuria, and both of those developed de novo nephrocalcinosis (one overt and one early-stage) at ultrasounds done 6 months after switching to burosumab.

Using age-specific SI thresholds, no child was hypercalciuric at Washout or Day 14; two children had ≥1 hypercalciuria episode later in follow-up. Mixed-model repeated-measures analysis of log10(×ULN) found no overall time effect from Washout through Day 56 (F(4,29) = 1.66, *p* = 0.185). Ratios of geometric means (×) vs Washout were: Day 14 1.06, Day 28 1.57, Day 42 2.52, Day 56 1.89 (Holm-adjusted *p*-values not significant). Median ×ULN remained <1.0 at all post-conversion visits. Two children exhibited de novo ultrasound changes consistent with nephrocalcinosis: one confirmed grade IIa and one possible/unconfirmed case.

**Fig. 3:**
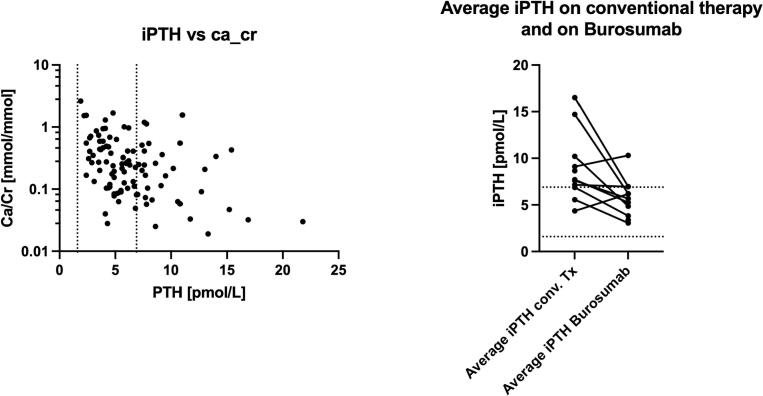
(A) The relationship between the intact PTH levels and the urinary calcium/creatinine ratio. (B) iPTH drops after conversion. Panel B includes the average PTH levels before and after starting burosumab, which explains the significantly lower number of observations.

Correlation analysis revealed no association between 1,25(OH)₂D and Ca:Cr and no association between TmP/GFR and Ca:Cr. A larger decline in intact PTH tracked with higher Ca:Cr (hypothesis-generating, Fig. 3).

Importantly, the two patients with the most marked drop in intact PTH developed mild nephrocalcinosis. Patient-level nephrocalcinosis data, including mutation type and imaging findings, are summarized in Table 2. In one of these patients, follow-up ultrasound confirmed resolution.

**Table 2:** n: Patients, PHEX gene mutation, age and onset of burosumab therapy, nephrocalcinosis status, and Renal and Bladder Ultrasound (RBUS) results.

Patient ID	Sex	PHEX mutation	Age at burosumab start (y)	Nephrocalcinosis status (study period)	RBUS summary
1	M	c.1753G>A	1.2	Pre-existing	Nephrocalcinosis on external ultrasound before burosumab
2	M	c.1753G>A	3.3	Pre-existing	Nephrocalcinosis on external imaging before burosumab (possible medullary sponge kidney)
3	F	c.1645+1G>A	8.4	Pre-existing	Significant medullary nephrocalcinosis before burosumab
4	M	exon 2 deletion	2.7	None	No nephrocalcinosis on imaging before and during study period
5	F	p.I204del	11	None	Multiple negative ultrasounds before burosumab
6	M	exon 12–13 deletion	5.1	None	Multiple negative ultrasounds before burosumab
7	M	exon 12–13 deletion	7.8	None	Multiple negative ultrasounds before burosumab
8	F	c.1753G>A	1.3	None	No nephrocalcinosis on RBUS before burosumab
9	F	c.1753G>A	0.6	Unknown	No imaging available during study period
10	F	c.1753G>A	3.7	Possible/unconfirmed	Punctate echogenic foci observed after burosumab; not seen on follow-up
11	F	c.1753G>A	1	De novo, confirmed	Grade IIa on external scan and verified
12	M	Xp22.11 deletion	16	Unknown	Negative RBUS post-burosumab within study period
13	F	p.Trp403*	6	None	Negative RBUS post-burosumab within study period

### 3.5 Adjunctive therapy and nephrocalcinosis prevention

Two additional children —converted after the study period—were started on hydrochlorothiazide plus potassium citrate at initiation. Normocalciuria was maintained, and no nephrocalcinosis developed; therapy was tapered after iPTH normalized.

## 4 Discussion

This study provides new insights into the renal safety of burosumab in pediatric XLH, extending the findings of Imel et al. (2019), Carpenter et al. (2018), and Whyte et al. (2019). Pediatric trials demonstrated superiority vs conventional therapy, whereas adult trials were placebo-controlled; neither systematically investigated hypercalciuria/nephrocalcinosis.

Our cohort revealed that one patient developed obvious new onset nephrocalcinosis following conversion to burosumab and one had echoic spots that may resemble beginning nephrocalcinosis while two children had ≥1 episode of hypercalciuria. This is a novel observation in long-term real-world data. Although previous authors focused on hypercalciuria during conventional therapy due to phosphate and calcitriol-induced renal calcium loss (Portale et al., 2024), our results suggest a different mechanism might be involved during burosumab therapy. These findings suggest that the magnitude of iPTH suppression, rather than absolute 1,25(OH)₂D or burosumab dose, may drive urinary calcium excretion; in our cohort, a larger iPTH decline tracked with higher Ca:Cr. It is possible that prior hyperparathyroidism may predispose to nephrocalcinosis. However, a rapid decline in iPTH post-burosumab may also play a role in altered calcium handling, warranting further study.

In a large cohort of XLH patients, nephrocalcinosis affected one-quarter of children and one-third of adults. It was associated with a higher prevalence of reduced eGFR in children (25 % vs 11 %), suggesting early renal impairment (Portale et al., 2024). Although most affected patients maintain renal function, a subset may progress to chronic kidney disease, underscoring the importance of ongoing monitoring.

Interestingly, despite the expected rise in 1,25(OH)_2_D after FGF23 inhibition, we found no correlation between its levels and urinary calcium excretion. These findings raise the hypothesis that abrupt PTH suppression may influence calcium handling in the distal nephron, warranting further investigation in larger studies. However, spot serum 1,25(OH)₂D levels may not reflect peak-trough variability across the dosing cycle, and this is a study limitation.

Notably, no further nephrocalcinosis developed in patients treated prophylactically with hydrochlorothiazide and potassium citrate, supporting the idea that hypercalciuria may be a modifiable risk factor.

Our findings align with the concerns raised by Harada et al., Goodyer et al., and Reusz et al. regarding nephrocalcinosis risk in XLH but extend the evidence to patients under burosumab therapy (Harada et al., 2021; Goodyer et al., 1987; Reusz et al., 1990). Schindeler et al. (2020) highlighted the importance of long-term follow-up studies to address concerns for hypercalciuria and nephrocalcinosis among pediatric patients with XLH undergoing burosumab therapy. We recommend ultrasound monitoring at baseline, 6 months after burosumab initiation, and annually thereafter, unless earlier changes in biochemistry warrant repeat imaging. Given current resource limitations, more frequent ultrasound screening may not be feasible in all centers. The absence of robust data on this complication highlights the urgent need for prospective trials.

The study is limited by its retrospective design and relatively small sample size, which is common in rare disease research. While the drop of PTH concentrations correlated with the degree of increase of the urinary calcium/creatinine ratio and development of nephrocalcinosis, the small sample size may have masked the statistical significance of the 1,25(OH)_2_D levels. We lacked standardized grading for all patients; one de novo case was graded IIa. Moreover, the spot calcium/creatinine ratio is less informative than the 24-hour calcium excretion, which forms an additional limitation. Additional limitations include the use of spot (non-fasting) urine samples, unmeasured dietary sodium intake (a major driver of within-child Ca:Cr variability), variable timing of blood sampling relative to dosing, and no global multiplicity correction across secondary outcomes. Nevertheless, the detailed longitudinal biochemical and imaging data strengthen our conclusions.

## 5 Conclusions

In conclusion, while burosumab remains the standard of care for XLH, clinicians should monitor patients closely for hypercalciuria and nephrocalcinosis, especially during the early phase of therapy. The limited sample size precludes definitive conclusions, and our findings should be considered hypothesis-generating.

## Abbreviations

1,25(OH)_2_D: 1,25 dihydroxyvitamin D
PHEX: Phosphate-regulating endopeptidase homolog X-linked, also known as phosphate-regulating gene with homologies to endopeptidases
SEM: standard error of the mean
TmP/GFR: ratio of tubular maximum reabsorption rate of phosphate to glomerular filtration rate
TRP: fractional tubular reabsorption of phosphate
ULN: upper limit of normal
XLH: X-linked hypophosphatemic rickets

## CRediT authorship contribution statement

**Guido Filler:** Writing – review & editing, Writing – original draft, Visualization, Validation, Supervision, Resources, Project administration, Methodology, Investigation, Formal analysis, Data curation, Conceptualization. **Harry Chandrakumaran:** Validation, Software, Resources, Methodology, Data curation. **Funmbi Babalola:** Writing – review & editing, Validation, Software, Resources, Methodology, Investigation. **Dougenie Emile:** Writing – review & editing, Methodology, Formal analysis. **Shih-Han Susan Huang:** Writing – review & editing, Validation, Supervision, Methodology, Investigation. **Robert Stein:** Writing – review & editing, Validation, Resources, Conceptualization.

## Informed consent

The manuscript was prepared and conducted ethically in accordance with the World Medical Association Declaration of Helsinki. The study was approved by the local ethics research board (file number 118344, PI G Filler, last approved 03-May-2022). Although the ethics committee waived the need for informed consent, each family consented verbally to this study.

## Grants or fellowships

None.

## Funding

No funding was available for this study.

## Declaration of competing interest

G Filler is a consultant for Kyowa Kirin and received a speaker honorarium from Kyowa Kirin Inc.

## Data Availability

Data will be made available on request.

## References

[bb0005] Ali D.S., Carpenter T.O., Imel E.A., Ward L.M., Appelman-Dijkstra N.M., Chaussain C., de Beur S.M.J., Florenzano P., Abu Alrob H., Aldabagh R., Alexander R.T., Alsarraf F., Beck-Nielsen S.S., Biosse-Duplan M., Crowley R.K., Dandurand K., Filler G., Friedlander L., Fukumoto S., Gagnon C., Goodyer P., Grasemann C., Grimbly C., Hussein S., Javaid M.K., Khan S., Khan A., Lehman A., Lems W.F., Lewiecki E.M., McDonnell C., Mirza R.D., Morgante E., Morrison A., Portale A.A., Rao C., Rhee Y., Rush E.T., Siggelkow H., Tetradis S., Tosi L., Guyatt G., Brandi M.L., Khan A.A. (2025 Jun 17). X-linked hypophosphatemia management in children: an international working group clinical practice guideline. J. Clin. Endocrinol. Metab..

[bb0010] Arango Sancho P. (2020). Complications of phosphate and vitamin D treatment in X-linked hypophosphataemia. Adv. Ther..

[bb0015] Brodehl J., Krause A., Hoyer P.F. (1988). Assessment of maximal tubular phosphate reabsorption: comparison of direct measurement with the nomogram of Bijvoet. Pediatr. Nephrol..

[bb0020] Carpenter T.O., Whyte M.P., Imel E.A., Boot A.M., Högler W., Linglart A., Padidela R., Van’t Hoff W., Mao M., Chen C.Y., Skrinar A., Kakkis E., San Martin J., Portale A.A. (2018). Burosumab therapy in children with X-linked hypophosphatemia. N. Engl. J. Med..

[bb0025] Colares Neto G.P., Yamauchi F. Ide, Hueb Baroni R., de Andrade Bianchi M., Cavalanti Gomes A., Chammas M.C., Matsunaga Martin R. (2019). Nephrocalcinosis and nephrolithiasis in X-linked hypophosphatemic rickets: diagnostic imaging and risk factors. J. Endocr. Soc..

[bb0030] Filisetti D., Ostermann G., von Bredow M., Strom T., Filler G., Ehrich J., Pannetier S., Garnier J.M., Rowe P., Francis F., Julienne A., Hanauer A., Econs M.J., Oudet C. (1999). Non-random distribution of mutations in the PHEX gene, and under-detected missense mutations at non-conserved residues. Eur. J. Hum. Genet..

[bb0035] Gohil A., Imel E.A. (2019). FGF23 and associated disorders of phosphate wasting. Pediatr. Endocrinol. Rev..

[bb0040] Goodyer P.R., Kronick J.B., Jequier S., Reade T.M., Scriver C.R. (1987). Nephrocalcinosis and its relationship to treatment of hereditary rickets. J. Pediatr..

[bb0045] Harada D., Ueyama K., Oriyama K., Ishiura Y., Kashiwagi H., Yamada H., Seino Y. (2021). Switching from conventional therapy to burosumab injection has the potential to prevent nephrocalcinosis in patients with X-linked hypophosphatemic rickets. J. Pediatr. Endocrinol. Metab..

[bb0050] Imel E.A., Glorieux F.H., Whyte M.P., Munns C.F., Ward L.M., Nilsson O., Simmons J.H., Padidela R., Namba N., Cheong H.I., Pitukcheewanont P., Sochett E., Hogler W., Muroya K., Tanaka H., Gottesman G.S., Biggin A., Perwad F., Mao M., Chen C.Y., Skrinar A., San Martin J., Portale A.A. (2019). Burosumab versus conventional therapy in children with X-linked hypophosphataemia: a randomised, active-controlled, open-label, phase 3 trial. Lancet.

[bb0055] Insogna K.L., Briot K., Imel E.A., Kamenický P., Ruppe M.D., Portale A.A., Weber T., Pitukcheewanont P., Cheong H.I., Jan de Beur S., Imanishi Y., Ito N., Lachmann R.H., Tanaka H., Perwad F., Zhang L., Chen C.Y., Theodore-Oklota C., Mealiffe M., San Martin J., Carpenter T.O., Randomized A., Double-Blind Placebo-Controlled (2018). Phase 3 trial evaluating the efficacy of burosumab, an anti-FGF23 antibody, in adults with X-linked hypophosphatemia: week 24 primary analysis. J. Bone Miner. Res..

[bb0060] Marra G., Taroni F., Berrettini A., Montanari E., Manzoni G., Montini G. (2019). Pediatric nephrolithiasis: a systematic approach from diagnosis to treatment. J. Nephrol..

[bb0065] Portale A.A., Carpenter T.O., Brandi M.L., Briot K., Cheong H.I., Cohen-Solal M., Crowley R., Jan De Beur S., Eastell R., Imanishi Y., Imel E.A., Ing S., Ito N., Javaid M., Kamenicky P., Keen R., Kubota T., Lachmann R., Perwad F., Pitukcheewanont P., Ralston S.H., Takeuchi Y., Tanaka H., Weber T.J., Yoo H.W., Zhang L., Theodore-Oklota C., Mealiffe M., San Martin J., Insogna K. (2019). Continued beneficial effects of burosumab in adults with X-linked hypophosphatemia: results from a 24-week treatment continuation period after a 24-week double-blind placebo-controlled period. Calcif. Tissue Int..

[bb0070] Portale A.A., Ward L., Dahir K., Florenzano P., Ing S.W., Jan de Beur S.M., Martin R.M., Meza-Martinez A.I., Paloian N., Ashraf A., Dixon B.P., Khan A., Langman C., Chen A., Wang C., Roberts M.S., Tandon P.K., Bedrosian C., Imel E.A. (2024). Nephrocalcinosis and kidney function in children and adults with X-linked hypophosphatemia: baseline results from a large longitudinal study. J. Bone Miner. Res..

[bb0075] Raeder H., Shaw N., Netelenbos C., Bjerknes R. (2008). A case of X-linked hypophosphatemic rickets: complications and the therapeutic use of cinacalcet. Eur. J. Endocrinol..

[bb0080] Reusz G.S., Hoyer P.F., Lucas M., Krohn H.P., Ehrich J.H., Brodehl J. (1990). X linked hypophosphataemia: treatment, height gain, and nephrocalcinosis. Arch. Dis. Child..

[bb0085] Schindeler A., Biggin A., Munns C.F. (2020). Clinical evidence for the benefits of Burosumab therapy for X-linked hypophosphatemia (XLH) and other conditions in adults and children. Front. Endocrinol..

[bb0090] Thacher T.D., Fischer P.R., Pettifor J.M., Lawson J.O., Manaster B.J., Reading J.C. (2000). Radiographic scoring method for the assessment of the severity of nutritional rickets. J. Trop. Pediatr..

[bb0095] Whyte M.P., Carpenter T.O., Gottesman G.S., Mao M., Skrinar A., San Martin J., Imel E.A. (2019). Efficacy and safety of burosumab in children aged 1-4 years with X-linked hypophosphataemia: a multicentre, open-label, phase 2 trial. Lancet Diabetes Endocrinol..

